# Maternal and infant antiretroviral therapy adherence among women living with HIV in rural South Africa: a cluster randomised trial of the role of male partner participation on adherence and PMTCT uptake

**DOI:** 10.1080/17290376.2020.1863854

**Published:** 2021-02-28

**Authors:** Deborah L. Jones, Violeta J. Rodriguez, Manasi Soni Parrish, Tae Kyoung Lee, Stephen M. Weiss, Shandir Ramlagan, Karl Peltzer

**Affiliations:** a Department of Psychiatry and Behavioral Sciences, University of Miami Miller School of Medicine, Miami, FL, USA; b Department of Psychology, University of Georgia, Athens, GA, USA; c Department of Public Health Sciences, University of Miami Miller School of Medicine, Miami, FL, USA; d HIV/AIDS/STIs and TB (HAST) Research Programme, Human Sciences Research Council, Pretoria, South Africa; e Department for Management of Science and Technology Development, Ton Duc Thang University, Ho Chi Minh City, Vietnam; f Faculty of Pharmacy, Ton Duc Thang University, Ho Chi Minh City, Vietnam

**Keywords:** HIV, women, adherence, male involvement, South Africa

## Abstract

‘Mother-to-child transmission of HIV’ can occur during the period of pregnancy, childbirth, or breastfeeding. ‘Prevention of mother-to-child transmission of HIV’ (PMTCT) in Mpumalanga Province, South Africa, is especially vital as the prevalence of HIV is 28.2% in women aged 15–49. PMTCT interventions resulted in a drop of MTCT rates in Mpumalanga from ∼2% in 2015 to 1.3% in 2016. This randomised controlled trial in Mpumalanga examined the potential impact of a lay healthcare worker administered intervention, ‘Protect Your Family’, on maternal and infant adherence, and to assess the relative influence of male partner involvement on infant and maternal adherence. This cluster randomised controlled trial used a two-phase and two-condition (experimental or control) study design where participants (*n* = 1399) did assessments both during pregnancy and post-postpartum. Only women participated in Phase 1, and both female and male partners participated in Phase 2. Results indicated that male involvement was associated with self-reported maternal or infant antiretroviral therapy (ART) adherence, but the intervention was not associated with ART adherence. Self-reported adherence was associated with depression, age, and partner HIV status. The study results provide support for the involvement of men in the antenatal clinic setting during pregnancy. Results also support further research on the meaning and assessment of male involvement and clarification of the constructs underlying the concept in the sub-Saharan African context. Outcomes provide support for male involvement and treatment of depression as adjuncts to improve uptake of both maternal and infant medication as part of the PMTCT protocol.

## Introduction

Mother-to-child transmission (MTCT) of HIV can occur during the period of pregnancy, childbirth, or breastfeeding. Worldwide, as of 2015, there were ∼1.8 million children (<15 years) living with HIV, with 150,000 new cases annually (CDC, 2018; UNAIDS, 2016). Prevention of MTCT of HIV (PMTCT) has been specifically targeted in South Africa, a country with one of the highest HIV prevalence worldwide (Avert, 2020). In 2016, there were ‘7,100,000 people living with HIV in South Africa’ (Council, 2016). Of these, 86% were aware of their diagnosis, 65% were receiving antiretroviral therapy (ART), and 81% had achieved viral suppression (Avert, 2020; Council, 2016). In Mpumalanga Province, South Africa, the HIV prevalence in women of reproductive age (15–49) is 28.2% (Council, 2016). In 2014, the South African PMTCT guidelines were revised so that all ‘pregnant or breastfeeding women with HIV’, would receive lifelong ART upon diagnosis; currently, more than 95% of pregnant women are accessing treatment in South Africa (UNAIDS, 2015). All neonates with any previous exposure to HIV were to be tested with PCR, and neonatal prophylaxis with daily nevirapine was recommended for 6 weeks (Hurst, Appelgren, & Kourtis, 2015). PMTCT interventions resulted in a drop of MTCT rates in Mpumalanga from ∼2% in 2015 to 1.3% in 2016 (Council, 2016). Despite these achievements, challenges to PMTCT in Mpumalanga remain, including variable clinic data, uneven condom distribution, delayed PCR results, eventuating in delayed commencement of infant ART, and inconsistent male circumcision delivery and uptake (Rodriguez et al., 2017).

Commencing ART in all pregnant or breastfeeding women and continuing lifelong treatment have been associated with improved outcomes for mothers, as well as decreased mother-to-child and women-to-partner transmission of HIV (WHO, 2015). Without any preventative measures, MTCT rates range from 15% to 45% (WHO, 2015). Interventions during pregnancy, childbirth, and breastfeeding have reduced this rate to below 5%, and in women receiving ART and exclusively breastfeeding to 6 months, transmission risk can be reduced to 0–1% (WHO, 2015). These interventions have included lifelong antiretroviral therapy, antiretroviral prophylaxis, caesarean section prior to labour and ruptured membranes, and total avoidance of breastfeeding. However, maternal adherence antiretrovirals may be reduced by stigma and discrimination within the health infrastructure and community, delayed prenatal care, socioeconomic challenges, less education, and home delivery (Prudden et al., 2017; Woldesenbet et al., 2015), as well as low community and partner support, healthcare staff shortages, limited access to healthcare facilities, and denial, depression, fear (Gourlay, Birdthistle, Mburu, Iorpenda, & Wringe, 2013). Stigma may also act as a barrier to adherence because it may hinder women from disclosing their HIV status to their partner (Sweeney & Vanable, 2016). In addition, comparisons of self-reported adherence to biological evidence (dried blood spots) have found women to overestimate compliance (Alcaide et al., 2017), suggesting that blood testing may provide a more accurate evaluation of adherence (Calcagno et al., 2015).

Male involvement during and after pregnancy is linked to positive outcomes for both maternal and infant health in low- and middle-income countries (Matseke et al., 2017; Yargawa & Leonardi-Bee, 2015). Broadly, male involvement is associated with a reduced maternal depression, fewer complications during childbirth, with greater use of healthcare services (Yargawa & Leonardi-Bee, 2015). While prevention of mother-to-child transmission (PMTCT) of HIV is largely dependent on maternal adherence to antiretroviral prophylaxis, it is also heavily influenced by the longitudinal support of male partners (Elias, Mmbaga, Mohamed, & Kishimba, 2017), given that men are the primary decision makers in low- and middle-income countries. When male partners are involved, the overall risk of vertical transmission of HIV and subsequent infant mortality drops by 40% (Aluisio et al., 2011).

Adherence interventions in South Africa include lay healthcare worker promotions, involvement in community and faith-based organisations, peer support groups (e.g. mothers-to-mothers*)*, and community-driven education and communication (Schmitz et al., 2019). Weekly reminders delivered by short text messages have also shown success by improving ART adherence by 13–16% in Kenya, though such interventions are limited by technology access (Pop-Eleches et al., 2011). Among infants breastfed by mothers with HIV, both mothers and infants must adhere to ART to prevent MTCT, though few studies have investigated adherence among infants under 12 months of age. Interventions tested to enhance paediatric adherence have mostly been conducted with small samples and in the U.S. (Haberer & Mellins, 2009). In addition, depression and intimate partner violence (PV), frequently observed in South Africa, have been associated with poor ART adherence in both mothers and infants, suggesting that targeted treatment of maternal depression and ART compliance may further reduce MTCT rates (Cook, Peltzer, Weiss, Rodriguez, & Jones, 2018). As such, this randomised controlled trial in rural Mpumalanga, South Africa, examined the potential impact of a lay healthcare worker administered intervention, ‘Protect Your Family (PYF)’, on maternal and infant adherence, accounting for this synergy of factors (i.e. depression, disclosure, stigma, and knowledge) influencing maternal and infant adherence. In addition, this study examined the relative influence of male partner involvement, theorising that men’s involvement during pregnancy could improve both infant and maternal adherence.

## Methods

### Study design

The study aimed to measure the effect of male involvement on the improvement of PMTCT. The study design was a two-phase and two-condition (experimental or control) cluster randomised controlled trial. In Phase 1 only women participated, while in Phase 2 both female and male partners. After randomisation, baseline assessments were completed between 6 and 30 weeks of pregnancy and participants were invited to participate in four group intervention sessions (or time-equivalent control sessions) antenatally and two post-partum individual sessions, led by lay healthcare workers. Women were re-assessed antenatally at 32 weeks of pregnancy, and postnatally at 6 weeks, 6 and 12 months. Retention at 12 months postnatal assessments in Phase 1 was 60% and Phase 2 74% (see complete trial diagram at Peltzer et al., 2017).

### Ethics approval

Ethics approval for this study was granted by the Human Sciences Research Council (HSRC) Research Ethics Committee (REC), protocol approval number REC4/21/08/13. Furthermore, additional approval was also obtained from the Department of Health and Social Welfare, Mpumalanga Provincial Government, and the University of Miami Miller School of Medicine Institutional Review Board (IRB ID: 20130238). Written informed consent was obtained from all participants.

### Recruitment

*Community health centres* (CHCs) in two health districts (Nkangala and Gert Sibande) in the province of Mpumalanga were assessed for study participation with the Provincial Department of Health of Mpumalanga. CHCs meeting the criteria for PMTCT sites in South Africa were eligible (National Department of Health - Republic of South Africa, 2015).

*Participants.* Candidates were HIV positive pregnant women, 18 years and older, and had a male partner. They were recruited from 10 April 2014 to 30 January 2017. Only in Phase 2, male partners were recruited and took part in sessions. Participants completed assessments using ‘audio computer-assisted self-interview (ACASI)’ in the language preferred by respective participants (English, isiZulu, seSotho) after providing informed consent (Peltzer et al., 2017).

### Randomisation and blinding

Twelve CHCs with MTCT rates in the upper 50th percentile of MTCT rates at study onset (>13%) were matched in a 1:1 ratio according to patient census and HIV rates. A randomisation programme was then used by the data manager to assign CHCs to experimental or control conditions; the other CHC was assigned to the opposite condition (more details, Jones, et al., 2014).

### Conditions

In the antenatal period, PYF sessions were held in a group format; post-partum sessions were conducted in individual or couples’ format.

*Intervention condition.* PYF is a behavioural PMTCT intervention administered by lay healthcare workers. ‘Intervention session 1: PMTCT, mutual HIV testing, stigma, disclosure and alcohol/drug use; Intervention session 2: communication, disclosure, adherence, male involvement and anxiety reduction; Intervention session 1–4: keeping the family safe at home and community, disclosure, intimate partner violence, partner involvement in pregnancy and birth; Intervention session 5: safe sex, family planning, immunisation, nutrition, adherence and anxiety reduction; Intervention session 6: family planning, immunisation, feeding, medication and anxiety reduction’ (more details, Peltzer et al., 2017, p. 4).

In Phase 2, men’s PYF sessions were intended for men with female partners with or without HIV, and covered issues on PMTCT for women, men’s and child health issues (Peltzer et al., 2017).

*Control condition* female and male participants viewed a time-matched group video presentation on ‘diarrhoea management, dehydration and exclusive breastfeeding, infant nutrition, immunisation and sexual abuse, followed by an individual or couples’ video presentation on fevers’ (Jones et al., 2014, p. 2669). Postnatally, video sessions included were on burns, and alcohol use (Peltzer et al., 2017).

*Training of lay healthcare workers and intervention quality assurance* have been described in detail previously (Jones et al., 2014). Briefly, the training was on the study protocol, informed consent, protection of human subjects, recruitment, assessment and use of ACASI technology, and the enhanced intervention condition staff attended a training course on the PMTCT protocol and use of cognitive behavioural (CB) intervention strategies in the intervention, etc.

### Measures

*Dried blood spot adherence.* TDF, EFV, and 3TC levels were measured among all study participants at 32 weeks of pregnancy. Adherence was defined as ‘detection of either 2 or 3 drugs (TDF, EFV, or 3TC). As FTC was not measured and the combination of TDF + FTC in a single pill is recommended, the combination of TDF + EFV detected was also deemed to be adherent. Non-adherence defined as no detectable ARV, one ARV detectable, or a combination of two ARVs detectable that did not include TDF + EFV (e.g. TDF + 3TC or 3TC + EFV)’ (Alcaide et al., 2017, p. 2137).

*Adult AIDS Clinical Trials Group (ACTG) Self-Reported Antiretroviral Adherence (4 days).* To assess maternal adherence at baseline, 32 weeks of pregnancy, and 6 weeks, 6 and 12 months post-partum, the ACTG (Chesney et al., 2000) self-reported antiretroviral (ARV) adherence measure was used. The ACTG asks about the number of ARV medications missed in the past 4 days. Responses were dichotomised into non-adherent if one dose was missed or adherent if no doses were missed.

*Mother-reported infant adherence to nevirapine prophylaxis*. To assess mother-administered infant adherence at 6 weeks, maternal self-report on the ACTG (Chesney et al., 2000) was used. In addition, mothers completed a 7-day ‘Visual Analog Scale (VAS)’. The VAS has participants self-rate their ARV adherence level ‘on a scale of 0 (*took none*) to 1 (*took half*) to 2 (*took all*) for each day in the past seven days’ (Giordano, Guzman, Clark, Charlebois, & Bangsberg, 2004).

*Demographic and psychosocial characteristics.* Demographic information evaluated included age, education, relationship status, income, history of HIV diagnosis of self, partner and children.

*Depressive symptoms*. The ‘Edinburgh Postnatal Depression Scale 10’ (EPDS-10) was utilised to calculate depressive symptoms, with a cut-off of 12 or more, at baseline (Cox, Holden, & Sagovsky, 1987) (*α* = 0.73).

*Disclosure.* An adapted Disclosure Scale (Visser, Neufeld, de Villiers, Makin, & Forsyth, 2008) addressed the disclosure of HIV status to sexual partners and family members.

*Male involvement.* An adapted male Involvement Index (Byamugisha, Tumwine, Semiyaga, & Tylleskar, 2010) assessed male participation during pregnancy (*α* = 0.83).

*Stigma.* The ‘AIDS-Related Stigma Scale (ARSS)’ (Kalichman et al., 2009) was utilised to assess HIV-related stigma (*α* = 0.74).

*Family planning knowledge.* Eight multiple choice questions adapted from the ‘Safer Conception Knowledge, Attitudes & Practices (SCKAP)’ and Family Planning Survey (Idonije, Oluba, & Otamere, 2011) were utilised to evaluate knowledge of family planning (*α* = 0.72).

### Statistical analyses

Means of continuous variables and proportions of categorical variables were used to describe participants demographically and psychosocially. To analyse attrition at follow-up, chi-square tests for categorical variables and *t*-tests for continuous variables were used. Variables associated with attrition at *p* < 0.01 were included as covariates. Male involvement was included in all models. Statistical significance was defined as *p* < 0.05.

To estimate the effect of demographic and psychosocial characteristics and the influence of phase and interventions on time-invarying outcomes (maternal 32-week adherence and longitudinal ACTG adherence), logistic regression models were used. Logistic regression was also used to assess the interaction effect of phase × intervention effects on infant ACTG and VAS adherence. To correct for data dependency (participants nested in community health centres), multi-level growth models were used. Odds ratios were utilised as effect sizes (Allen & Le, 2008). To incorporate the time-varying outcome (ACTG adherence), a multi-level growth model was constructed to evaluate the clinic-level variation for the condition effects on longitudinal changes (trajectories) in ACTG adherence. This model demonstrated that the condition effects at the clinic level was 0 (*p* = 0.999), signifying that all clinics had same effects per condition. As such, a multi-level structure for clinic was not needed, and a growth curve model was used instead.

To handle missing data across all models, a multiple imputation strategy was used, specifying ten imputed datasets (Asparouhov & Muthén, 2010). All analyses were performed using Mplus (version 8.1) on a Windows operating system (Muthén & Muthén, 2014).

## Results

### Baseline sample characteristics of women

Women were an average of 28 years of age (SD = 5.83), and 48% reported 10–11 years of education (see Table 1). All women had a partner, although only 26% were living with their partner and 20% were married. More than half (55%) of the women had been diagnosed with HIV during the current pregnancy, 50% reported that their pregnancy was unplanned, 79% had at least one child, 29% reported that their partner was HIV positive, and 4% reported to have an HIV positive child. Nearly half (45%) of women were above the clinically significant cut-off for depression,15% of women revealed having more than two alcoholic drinks in the 30 days, and 61% had disclosed their HIV status to their male partner. Two-thirds (69%) of women self-reported 4 days of adherence (ACTG) at baseline, and 76% were assessed as adherent by DBS at 32 weeks. At 6 weeks post-partum, 91% of women indicated 4 days of adherence (ACTG) to their infant’s medication regimen, and 89% reported 7 days of adherence (VAS) to their infant regimen.

**Table 1.** Sample at baseline and bivariate associations with attrition (*N* = 1399).

	Total (*N* = 1399)	Lost (*n* = 877)	Retained (*n* = 522)	*t/X^2^*, *p*
	*N* (%)	*N* (%)	*N* (%)	* *
	Mean (SD)	Mean (SD)	Mean (SD)	* *
**Sociodemographics**				
Mean age in years (SD)	28.45 (5.82)	28.19 (5.71)	28.90 (5.98)	**2.21, 0.027**
Education				
0–Grade 9	287 (20.5%)	175 (20.0%)	112 (21.5%)	
Grade 10–11	673 (48.1%)	432 (49.3%)	241 (46.2%)	
Grade 12 or more	439 (31.4%)	270 (30.8%)	169 (32.4%)	1.27, 0.529
Relationship status				
Unmarried, living separate	754 (53.9%)	484 (55.2%)	270 (51.7%)	
Unmarried, cohabiting	368 (26.3%)	219 (25.0%)	149 (28.5%)	
Married	277 (19.8%)	174 (19.8%)	103 (19.7%)	2.34, 0.314
Work				
Unemployed	1072 (76.6%)	680 (77.5%)	392 (75.1%)	
Employed	327 (23.4%)	197 (22.5%)	130 (24.9%)	1.09, 0.297
Monthly income				** **
< 1000 Rand (USD$70)	498 (35.6%)	348 (39.7%)	150 (28.7%)	** **
≥ 1000	901 (64.4%)	529 (60.3%)	372 (71.3%)	**17.10, < 0.001**
**Health variables**				
Dagnosed with HIV during this pregnancy				
No	620 (45.3%)	373 (43.4%)	247 (48.4%)	
Yes	749 (54.7%)	486 (56.6%)	263 (51.6%)	3.24, 0.072
Pregnancy unplanned				
No	682 (49.8%)	426 (49.6%)	256 (50.2%)	
Yes	687 (50.2%)	433 (50.4%)	254 (49.8%)	0.05, 0.829
Number of children				
None	298 (20.8%)	198 (22.6%)	100 (19.2%)	
One or more	1101 (78.7%)	679 (77.4%)	422 (80.8%)	2.28, 0.131
Partner HIV positive				** **
No/unknown	1020 (73.0%)	664 (75.7%)	356 (68.5%)	** **
Yes	377 (27.0%)	213 (24.3%)	164 (31.5%)	**8.71, 0.003**
Has HIV positive child				
No	1032 (93.8%)	647 (95.3%)	385 (91.4%)	
Yes	68 (6.2%)	32 (4.7%)	36 (8.6%)	1.35, 0.245
Time since ART initiation (months)	15.25 (25.37)	13.30 (23.77)	18.52 (27.57)	**4.10, < 0.001**
Time since HIV diagnosis (months)	24.15 (35.94)	22.14 (34.82)	27.55 (37.54)	**3.25, 0.001**
**Psychosocial variables**	** **	** **	** **	** **
Male involvement	7.30 (3.19)	7.30 (3.22)	7.28 (3.14)	0.33, 0.745
Stigma	1.27 (1.27)	1.31 (1.34)	1.22 (1.14)	0.55, 0.552
Family planning knowledge	4.48 (1.31)	4.45 (1.34)	4.53 (1.25)	**2.15, 0.032**
Depression	11.73 (6.01)	12.18 (5.93)	10.98 (6.07)	**3.65, < 0.001**
Psychological Intimate Partner Violence	3.46 (6.02)	3.27 (5.99)	3.78 (6.07)	1.75, 0.080
Physical Intimate Partner Violence	1.26 (4.16)	1.23 (4.28)	1.31 (3.93)	0.43, 0.671
Alcohol (> 2 drinks in a day in the past month)				
No	1171 (85.5%)	740 (86.0%)	431 (84.5%)	
Yes	199 (14.5%)	120 (14.0%)	79 (15.5%)	0.61, 0.435
Disclosure of HIV status to Partner				
No	539 (39.3%)	352 (40.9%)	187 (36.7%)	
Yes	831 (60.7%)	508 (59.1%)	323 (63.3%)	2.44, 0.118
**Adherence variables**	** **	** **	** **	** **
Maternal DBS Adherence (32 weeks of pregnancy)				** **
Nonadherence	136 (24.0%)	52 (20.0%)	84 (27.4%)	** **
Adherence	431 (76.0%)	208 (80.0%)	223 (72.6%)	**4.18, 0.041**
Maternal ACTG 100% 4-day Adherence				
Nonadherence	429 (31.3%)	267 (31.1%)	162 (31.8%)	
Adherence	940 (68.7%)	592 (68.9%)	348 (68.2%)	0.07, 0.793
Infant 100% 4-day ACTG Adherence				
Nonadherence	68 (8.7%)	40 (7.7%)	28 (10.7%)	
Adherence	711 (91.3%)	477 (92.3%)	234 (89.3%)	1.90, 0.168
Infant 100% 7-day VAS Adherence				
Nonadherence	89 (11.4%)	54 (10.4%)	35 (3.3%)	
Adherence	690 (88.6%)	463 (89.6%)	227 (86.7%)	1.46, 0.227

### Eligibility and recruitment

In all, *N* = 3500 women were screened and *n* = 2,791 in Phase 1, and *N* = 2204 were screened and *n* = 1489 were not eligible in Phase 2. The primary reasons for ineligibility to participate included not being HIV-infected, not being pregnant, not having a male partner, being 32 weeks pregnant, not being at least 18 years of age, currently participating in another study, or lack of time to participate.

### Attrition analyses

As seen in Table 1, attrition analyses indicated that women who were retained were more likely to be older, have higher income, have an HIV-infected partner, have longer time since ART initiation and HIV diagnosis, greater family planning knowledge, and greater depressive symptoms. In Phase 1, retention from baseline to 32-weeks was 61%, 47% from 32-week to 6-week postnatally, and 50–60% at 6- and 12-month follow-up. In Phase 2, retention from baseline to 32 weeks was 68%, 63% from 32-week to 6-week postnatally, and 69–74% at 6- and 12-month follow-up.

### Associations with adherence

*Maternal 32-week dried blood spot adherence.* Neither phase nor condition was associated with maternal adherence by DBS at 32 weeks; the interaction between phase and condition was also not statistically significant. Less family planning knowledge at baseline predicted DBS adherence at 32 weeks of pregnancy (AOR = 0.856 [0.741, 0.989]) (see Table 2).

**Table 2.** Intervention effects and on maternal dry blood spot (DBS) adherence at 32 weeks (*N* = 567).

	Maternal DBS Adherence	
Variable	AOR [95% CI]	
*Fixed effects*		
Condition	0.580 [0.192, 1.749]	
Phase	0.761 [0.481, 1.205]	
Intervention × Phase	1.924 [0.715, 5.175]	
*Controls (at baseline)*		
Age	1.011 [0.98, 1.043]	
Monthly income	1.260 [0.853, 1.860]	
Partner HIV positive	1.724 [0.769, 3.867]	
Time since ART initiation	0.990 [0.976, 1.005]	
Time since HIV diagnosis	1.001 [0.992, 1.011]	
Family planning knowledge	0.856 [0.741, 0.989]*	
Depression	0.993 [0.967, 1.020]	
Male involvement	0.993 [0.942, 1.047]	
*Random effects*	B (SE)	95% CI
Intercept	−1.092	−3.298, 1.114
Adherence	0.013	−0.074, 0.101
*Model fit*		
−2LL (Deviance)	1483.71	
Numbers of parameters	13	
AIC/BIC	1509.713/1578.145	
ICC (without covariates)	0.041	

*Maternal longitudinal self-report of adherence.* As noted in Table 3, neither phase nor condition was associated with maternal ACTG adherence longitudinally. The interaction between phase and condition was also not statistically significant. Having an HIV-uninfected partner (AOR = 0.546 [0.331, 0.899]), and decreased depressive symptomatology (AOR = 0.925 [0.901, 0.949]) predicted ACTG adherence longitudinally.

**Table 3.** Predicting adherence longitudinally at baseline and 32 weeks of pregnancy, and 6 weeks, 6, and 12 months post-partum.

	Maternal 100% 4-day	
ACTG Adherence		
Variable	AOR [95% CI]	* *
*Fixed effects*	** **	** **
Condition	0.588 [0.212, 1.627]	
Phase	1.303 [0.841, 2.020]	
Intervention ×Phase	0.853 [0.455, 1.598]	
*Controls (at baseline)*		
Age	0.976 [0.948, 1.004]	
Monthly income	1.142 [0.825, 1.579]	
Partner HIV positive	0.546 [0.331, 0.899]*	
Time since ART initiation	0.997 [0.988, 1.005]	
Time since HIV diagnosis	0.997 [0.992, 1.003]	
Family planning knowledge	0.901 [0.811, 1.003]	
Depression	0.925 [0.901, 0.949]***	
Male involvement	1.041 [0.984, 1.100]	
*Random Effects*^a^	B (SE)	95% CI
Intercept (baseline)	−0.025 (0.038)	−0.099, 0.049
Adherence	0.002 (0.001)	0.001, 0.004
*Model fit*		
−2LL (Deviance)	7307.778	
Numbers of parameters	27	
AIC/BIC	7372.109/7503.464	
ICC (without covariates)	0.000	

*Infant 6-week reported adherence.* Phase 2 had a significantly greater proportion of women reporting adherence to their infants’ regimen (AOR = 3.045 [1.326, 6.991]) on the VAS (see Table 4). Decreased depressive symptoms at baseline predicted 4-days adherence to infant’s medication regimen at 6 weeks (ACTG; AOR = 0.949 [0.905, 0.995]), and 7 days of infant adherence (VAS; AOR = 0.941 [0.907, 0.975]).

**Table 4.** Intervention and phase effects on infant VAS and ACTG adherence at 6 weeks.

	Infant 100% 4-day	Infant 100% 7-day		
	ACTG Adherence	VAS Adherence		
	AOR [95% CI]	AOR [95% CI]		
*Fixed effects*				
Condition	1.992 [0.460, 8.619]	1.459 [0.369, 5.778]		
Phase	1.422 [0.678, 2.982]	3.045 [1.326, 6.991]**		
Intervention × Phase	0.466 [0.191, 1.133]	0.397 [0.154, 1.023]		
*Controls (at baseline)*				
Age	0.980 [0.937, 1.024]	0.996 [0.946, 1.049]		
Monthly income	1.104 [0.548, 2.225]	1.201 [0.665, 2.167]		
Partner HIV positive	0.860 [0.386, 1.918]	0.507 [0.230, 1.119]		
Time since ART initiation	1.006 [0.980, 1.032]	1.008 [0.994, 1.023]		
Time since HIV diagnosis	1.013 [0.995, 1.032]	0.993 [0.984, 1.002]		
Family planning knowledge	0.851 [0.687, 1.055]	0.980 [0.841, 1.142]		
Depression	0.949 [0.905, 0.995]*	0.941 [0.907, 0.975]**		
Male involvement	1.022 [0.935, 1.117]	0.952 [0.876, 1.034]		
*Random effects*	B (SE)	95% CI	B (SE)	95% CI
Intercept	3.757 (1.219)	−6.146, −1.367	−3.356 (0.951)	−5.219, −1.493
Adherence	0.031 (0.091)	−0.148, 0.210	0.031 (0.064)	−0.094, 0.157
*Model fit*				
−2LL (Deviance)	821.658	960.832		
Numbers of parameters	13	13		
AIC/BIC	847.658 / 916.091	986.832/1055.265		
ICC (without covariates)	0.068	0.104		

## Discussion

This randomised controlled trial examined the impact of the lay healthcare worker delivered intervention, Protect Your Family, on maternal and infant adherence, and the influence of male partner involvement on adherence outcomes in the context of the intervention. Male involvement, as measured by participation of men in the study, was associated with reported maternal or infant adherence, but contrary to expectation, the intervention was not associated with adherence. Similarly, adherence assessed by dried blood spot only associated with lower family planning knowledge, but not with male involvement or condition assignment.

Study results suggest male involvement, which was promoted in Phase 2 of the study, supported adherence by mothers for themselves and when providing medication to their infants, even when controlling for other factors associated with adherence, e.g. depression, partner HIV status, and age. Although the male involvement scale (Byamugisha et al., 2010) does not target specific factors predictive of adherence, the study design targeted increasing men’s involvement in the perinatal period, encouraging them to attend assessments and sessions in the antenatal clinics. As such, male involvement would likely have been stimulated by exposure to the clinic setting. Results promote the careful review of the elements of male involvement scales, as the elements included may not necessarily be the essential elements needed to enhance PMTCT. Previous research has shown that although male involvement is one of the most challenging components of PMTCT, it improves neonatal and maternal outcomes (Maonga, Mahande, Damian, & Msuya, 2016). However, in a review of several studies, male participation during the pregnancy period improved women’s knowledge of risk factors during pregnancy, but it did not influence birth preparedness, antenatal care utilisation, or the rate of miscarriages. Male involvement was also related to increased use of institutional delivery and skilled birth attendance, but did not have an association with neonatal outcomes. In addition, male involvement was related to increased uptake in the postnatal period, but had a minimal effect on breastfeeding and survival (Aguiar & Jennings, 2015). It is therefore possible that male involvement may be beneficial to women due to increased social support, but it may not be sufficient to influence neonatal and maternal health or increase PMTCT uptake. There are also systemic barriers to supporting male involvement in rural clinics, such as limited space, which may overshadow the positive benefits of promoting male partner participation (Rodriguez et al., 2017). Results support previous studies (Cook et al., 2018) underscore the relationship between depression and poor maternal adherence and suboptimal provision of medication to infants, as well as the relationship between maternal and infant adherence behaviours.

Several issues may have influenced study outcomes, the primary one being attrition in Phase 1 compared to Phase 2. Some of the reasons for drop-out from the study included miscarriages, infant deaths, or relocation. Because men in Phase 2 were required to participate in the study, it is possible that women in Phase 2 may have received more support from their partners to attend clinic appointments. Though the influence of attrition was controlled for, and data imputation was performed to respond to participants with missing data, participants may have self-selected to leave the study due to depression, recency of diagnosis, or partner HIV status. The study results may have also been influenced by the high prevalence of non-disclosure of HIV status from women to their partners (39%). Although the intervention was aimed at promoting women’s disclosure of HIV status, 55% of the women were diagnosed during the current pregnancy; such a recent diagnosis prior to study initiation may not have provided women with sufficient time to disclose it to others. Study results also underscore the conundrum which arises when self-reported adherence and biological measurement of adherence disagreed, such that biological measurement of adherence remains the gold standard for assessment (Calcagno et al., 2015). As such, results must be interpreted with caution, as the most compelling outcomes are those provided by self-report. Future studies should ensure the use of DBS and biological assessment across longitudinal time points. Finally, many clinics, in addition to provision of the PMTCT protocol, now include programmes targeting PMTCT (e.g. mothers-to-mothers) the combination of which may achieve a ceiling effect with regard to adherence and PMTCT uptake.

In conclusion, this study provides support for the involvement of men in the antenatal clinic setting, and results provide support for ensuring that men are welcomed in that traditionally female environment. Results also support further research on the meaning and assessment of male involvement and clarification of the constructs underlying the concept in the sub-Saharan African context. Finally, outcomes provide support for male involvement and treatment of depression as adjuncts to improve uptake of both maternal and infant medication as part of the PMTCT protocol.

## Footnotes

^a^
Random effects indicates the estimated variances from random effects regression model.
